# Multifaceted precarity: pandemic experiences of recent immigrant women in the accommodation and food services sector

**DOI:** 10.1186/s12889-023-17392-y

**Published:** 2023-12-13

**Authors:** Alice Mũrage, Julia Smith

**Affiliations:** Simon Fraser University, Burnaby, British Columbia Canada

**Keywords:** Work precarity, Mental health, COVID-19 pandemic, Recent immigrant women, Marginalization, Accommodation and food services sector

## Abstract

The COVID-19 pandemic disproportionately affected those who face historical and ongoing marginalization. In centering pandemic experience of recent immigrant women in the accommodation and food services sector in Canada, we examine how their precarious work translated to experiences of work precarity and wellbeing. This paper illuminates how pre-existing and ongoing marginalization are reproduced during a health crisis for those at the intersection of gender, race, migration, and labour inequities. Using semi-structured interviews and systematic analysis using the Work Precarity Framework, we found that the pandemic exacerbated pre-existing socio-economic marginalization and resulted in unique experiences of work precarity. The latter was experienced as precarity of work (unpredictable work hours and job or employment insecurity), precarity from work (inadequate incomes), and precarity at work (physical, psychological, and relational unsafety). Work precarity stood out as a social determinant of health in relation to its outcome of degraded mental health and wellbeing. Recognizing the role of policies in producing, reproducing, and distributing precarity, we recommend policy directions to reduce social inequities in pandemic recovery.

## Introduction

The COVID-19 pandemic and subsequent public health policies exacerbated experiences of precarity, particularly for those already experiencing livelihood insecurities due to economic and social marginalization. Precarity can denote conditions of employment, legal status, housing situations among other human conditions relating to access to societal resources. Just as various aspects of marginalization are intrinsically connected, these different avenues of precarity often intertwine. Employment precarity, as central to economic marginalization, looks differently based on context as it is shaped by “ever-changing realities of local labour markets” [1]. However, it is characterized by insecure employment arrangement including lack of a contract or defined employment term, part-time, temporary or casual work, low wages, and lack of benefits such as health coverage or paid sick leave [1]. Studies have evidenced precarious work as a social determinant of health highlighting direct consequence of occupational hazards [2], limited access to social and health benefits [3], and material deprivation due to low incomes [4], as well as indirect consequence such as social marginalization [5] and poor mental health [6].

Since 2020, scholars have articulated the growth of “pandemic precarities”, beginning to map how the pandemic response has interacted with existing precarities as well as created new ones [7–10]. For instance, women in Canada have been disproportionately impacted by the COVID-19 pandemic because of their over-representation in part-time or temporary work in the services sectors, precarious work arrangements and occupations vulnerable to economic downturns [11, 12], with recent immigrant women more impacted than Canadian-born women [13]. It has also been noted that essential workers are not spared from precarity as continued production of goods and services has been prioritized by governments, industries, and employers at the expense of workers, with worksites becoming “sacrifice zones” [14, 15]. However, there has been little analysis that explores the overlaps between pandemic precarities and pre-existing social determinants of health related to precarious work.

This paper contributes to the growing literature on pandemic precarities as well as on precarity as a social determinant of health by focusing on precarity experienced by recent immigrant women working within the Accommodations and Food Services (AFS) sector in British Columbia, Canada. We focused specifically on recent immigrant women recognizing intersecting marginalization likely faced by this demographic, with evidence identifying them as among those most effected by both COVID-19’s economic impacts and labour precarity [13]. We focus on the AFS sector as immigrant women are overrepresented in the sector, which is characterized by a high degree of precarity. In 2017, immigrants made up 25.8 percent of all workers in Canada but 34.7 percent of workers in the AFS sector [16]. Workers in this sector are also likely to be employed precariously [17]. The AFS employs disproportionately high numbers of part-time workers, 47.6 percent compared to 18.4 percent across all other sectors (see, Table 1). Women are overrepresented among part-time workers within the sector, at 61.5 percent [18]. Work in the sector became even more precarious during the pandemic. Employment in the sector fell by 20.8 percent between 2019 and 2021, representing a loss of 252,100 jobs [18], and part time work in the sector increased (see, Table 1).

**Table 1:** AFS Sector Labour Characteristics Compared to All Sectors in Canada

	**AFS Sector**		**All Sectors**	
	**2019**	**2021**	**2019**	**2021**
**Women workers**	56.2%	56.1%	47.5%	47.3%
**Youth workers**	41.8%	41.5%	13.6%	13.0%
**Full-time workers**	56.9%	52.4%	81.0%	81.6%
**Part-time workers**	43.1%	47.6%	19.0%	18.4%

Data on categories of workers calculated as a percentage of total workers (15 years & above)

Data Source: Statistics Canada, Labour Force Characteristics by Industry, annual (× 1,000) Table 14–10-0023–01, 2022

In documenting lived experiences of precarity of recent immigrant women working in this sector during the pandemic we aim to provide a systematic analysis, based on the Work Precarity Framework, of enablers, moderators, and outcomes of precarity and intersectional factors that contribute to the spectrum of precarity experienced during public health crises like the COVID-19 pandemic.

### Precarity

Precarity is not experienced equally and can be considered an indicator of societal inequities. It reflects uneven power relations along lines such as gender, race/ethnicity, and citizenship, and this varies depending on space–time context [19]. Through socio-economic policies (or lack thereof), governments play an important role in enabling and distributing precarity. For instance, Canadian migration policies on temporary foreign workers are noted to be a direct contributor to precarious work conditions of these workers [20]. Female Temporary Foreign Workers face additional structural violence associated with race and gender including risk of exploitation and abuse in the workplace [21]. Socio-economic marginalization such as that resulting from racism enable precarity [17].

Precarity has been defined as a condition of dependency [22], economic and social insecurity [23], vulnerability relative to contingency [19], and a sense of instability [24]. While precarity can denote conditions of employment, legal status, housing situations among other human conditions, in this paper, we are particularly concerned with precarity as it relates to employment. Precarious work is characterized by temporary work, involuntary part-time work, economic insecurity, low wages, lack of workplace protections and rights, and physically and psychologically unsafe workplaces among other conditions [1, 17, 25].

Precarity must be understood through an intersectional lens as legal status, citizenship, racialization, and gender are intricately linked to precarious work in the Canadian labour market. For instance, studies demonstrate that workers with undetermined legal status are more likely to be employed precariously, work conditions that typically persist even after the workers obtain secure status [26]. Other studies show that racialized workers are also more likely to be precariously employed compared to White workers [1, 27, 28]. Data also indicate that foreign-born workers are more likely to be precariously employed compared to Canadian-born workers; the former receiving a degree of protection after attaining citizenship [1, 28]. Data from 2018 reveal that while both women and men are affected by precarious work, this is experienced differently, with men enjoying an income advantage [29]. While women workers are more likely to be employed in low-pay and part-time jobs, men are more likely to be self-employed or employed in seasonal work [30]. Further, a recent study in British Columbia reveal that women are less likely than men to hold standard employment in the province [1]. These findings can partly be explained by the women’s overrepresentation in the service sector (which typically offer low wages and precarious work) and childcare responsibilities (which force women to take on part-time jobs) [30–32]. Indeed, social location, defined as “interaction between social relations, such as gender and ‘race,’ and political and economic conditions”, has been identified as among factors shaping precarious employment [25].

These studies indicate that foreign-born, non-citizen, racialized women workers would be worst off in the precarious work continuum. However, factors contributing to one’s experiences with precarious work are multiple and non-linear. Intersectionality scholars caution the difficulty in distinguishing which social identities and structural barriers contribute to overall lived experiences [33, 34]; in this case experiences in the labour market.

Precarious work contributes to poor health outcomes and is hence a driver of health inequities. While the study of precarious employment and the study of social determinants of health are fairly siloed (the former nestled in labour studies and the later in public health), increasing numbers of empirical studies demonstrate that work conditions and health outcomes are intrinsically linked. Social determinants of health (SDoH) frameworks highlight how various non-medical factors (i.e., living and working conditions) and policies shaping these conditions influence health outcomes [35]. Where employment conditions deviate from standard employment relationship, four components notably affect health outcomes: uncertainty/control (over access to work, influence over terms and conditions etc.) workload, support, and household insecurity (i.e., low and variable incomes) [25]. Precarious work not only operationalizes as a SDoH but also interacts with and contributes to other determinants such as access to healthcare services and housing and social insecurity.

Those at the intersections and experiencing multiple forms of oppression are therefore likely to face higher health inequities. One study found that while employment precarity was associated with poor self-reported physical and mental health, those in low income and less secure employment reported poorer health [1]. Immigrant and racialized workers are also more likely to experience poorer health outcomes. Recent immigrants, for instance, are less likely to raise health and safety concerns at work, compared to their non-immigrant colleagues [1]. A longitudinal quantitative study found that while new immigrants and migrants were healthier than Canadian-born residents at arrival, their health declined after immigration, with those who are racialized more affected [36]. Workers under the Temporary Foreign Workers (TFW) program face additional work and life precarities that impact both their physical and mental health, with women facing unique challenges associated with care giving roles [37].

### Work precarity framework

**Fig. 1:**
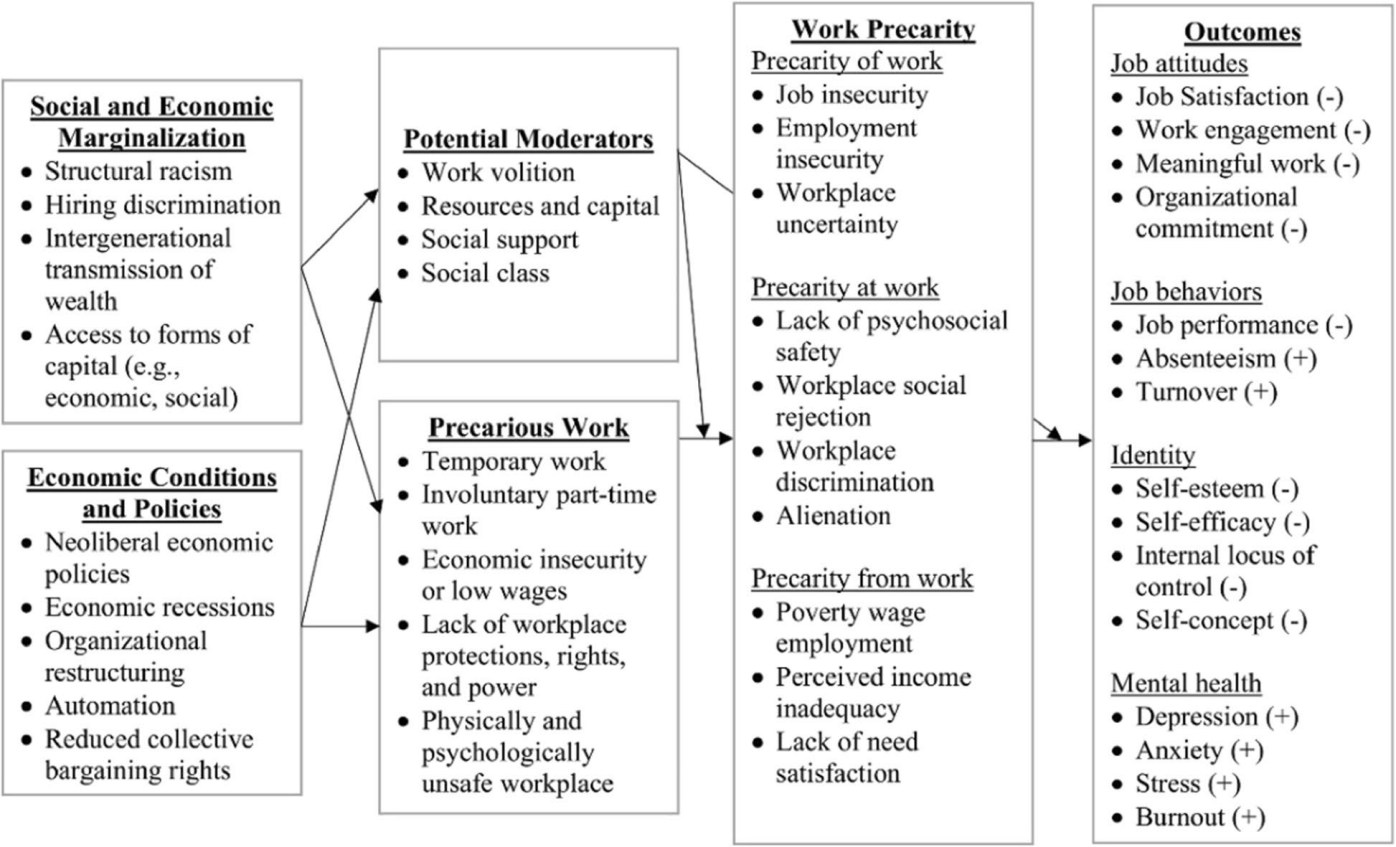
Work Precarity Framework by Allan, Autin, and Wilkins-Yel, 2021 Reprinted from Journal of Vocational Behaviour, vol. 126, Allan B. A., Autin, K. L. & Wilkins-Yel, K. G., Precarious work in the 21st century: A psychological perspective, 103491, 2021, with permission from Elsevier

We utilized the Work Precarity Framework to inform our analysis of pandemic precarities experienced by recent immigrant women in the AFS sector in Canada during the COVID-19 pandemic. This evidence-informed tool was developed by Allan et al. [17] to illustrate the functioning of work precarity. It illustrates how social and economic marginalization, economic conditions and policies, and potential moderators interact to shape experiences of work precarity and psychosocial outcomes for workers (see Fig. 1). The framework makes a distinction between precarious work as objective work conditions and work precarity as the subjective experience of precarious work. It highlights three experiences of precarity: uncertainty and insecurity about one’s immediate and long-term occupational future (precarity of work), safety and security at work (precarity at work), and consistent failure to meet basic needs (precarity from work).

The extent of precarious work’s manifestation in an individual’s life as work precarity is mediated by moderators such as access to resources and social support. An individual with savings, for instance, would not experience precarity from work in the same way as an individual without savings. Social and economic marginalization (e.g., structural racism) and economic conditions and policies play a central role in the distribution of precarious work, and consequently work precarity, and hence act as enablers of precarity. Conversely, they can also distribute potential moderators of precarious work and work precarity. The Work Precarity Framework outlines psychosocial outcomes of work precarity ranging from individual’s job attitudes and behaviours, sense of identity, and mental health. Moderators can, however, also mediate on the experience of these outcomes. An individual with a social support network, for instance, may be shield from negative mental health outcomes.

Our study utilized the Work Precarity Framework in its analysis of lived experiences of recent immigrant women workers to understand how precarious work shaped their experiences, and which enablers and moderators affected these experiences in the context of the COVID-19 pandemic. The framework was chosen for this purpose as it portrays the processes and factors than influence experiences of precarity. While employment precarity contributes to both physical and mental health outcomes, this framework focusses on the latter. As the framework is evidence-informed, this paper does not attempt to test or validate it, but applies it as a tool for analysis.

## Methods

This research aimed to explore lived experience of precarity of recent immigrant women working in the AFS sector in British Columbia Canada. We define women as anyone who identifies as such regardless of sex assigned at birth. The Canadian government defines immigrants as individuals who have been granted “the right to live in Canada permanently by immigration authorities” [38]. However, we acknowledge that temporary residents including temporary foreign workers and international students often end up living many years in Canada with many gaining permanent residence status. Transition from migrant to immigrant status is fairly common: In 2021, for instance, 168,656 temporary foreign workers transitioned to permanent residents [39]. Based on labour market needs, the government periodically supports this transition through programs such as the Interim Pathway for Caregivers and the Permanent Residence Pathway Program support this transition [40, 41]. We defined immigrants as individuals who move to Canada either through permanent or temporary status with an intention to making Canada their permanent home. Research participants came to Canada through a variety of pathways including as temporary foreign workers, refugees, and through spousal sponsored permanent residency. They all arrived in Canada ten years or less prior to the pandemic (since 2009) from Southern Asia, Eastern Asia, Middle East, North Africa, Central Africa, and Central America.

We conducted semi-structured interviews with 11 respondents between September and December 2021. Research participants were sampled by promoting recruitment through organizations supporting recent immigrants in the province. This approach allowed for culturally appropriate communication about the research. While this study is limited in sample size, likely a telltale of busy lives of this demographic,^1^ the lived experiences shared provide insights into, and questions around, broader trends identified elsewhere. Qualitative studies of a similar size have been found to contribute meaningful inquiry reflective of the experiences of those most affected in policy discussions [42–45].

Interview questions inquired on pandemic experiences relating to work, income and health. The semi-structured nature of this inquiry allowed participants to narrate other aspects of their pandemic experiences which were meaningful to them. Six of the eleven participants required language support during interviews. This was offered by women working with community organizations serving immigrants and hence understood the context of this work. To enhance the quality of data collection, careful instructions were provided to interpreters prior to interviews, interpreters took on a passive role (by not elaborating on the meaning of what participants said) mostly interpreting verbatim and researchers debriefed with interpreters after each interview [46]. Where interpreters used third-person narration, this was changed to first-person narration to reflect the voice of participants.

As a first step in data analysis, we familiarized ourselves with the data by reading interview transcripts and discussing grounding themes [47]. We subsequently used the Work Precarity Framework to structure our analysis, employing topical themes from the framework which corresponded to our initial themes as thematic codes. To better contextualize the experiences of workers in the AFS sector, we analysed industry-level data available through Statistics Canada and conducted two key informant interviews: Both key informants work with organizations (including one union) supporting workers’ rights in the hotels and restaurant industries, respectively. A key informant from a community organization supporting immigrants was also interviewed for insight on how such organizations moderate work precarity and related outcomes.

## Results

### Social and economic marginalization

Research participants highlighted unique challenges they faced, prior to the COVID-19 pandemic, as recent immigrants which made them vulnerable to precarious work and work precarity. Language barriers, lack of recognition of prior education, professional qualifications, and work experience, and limited social networks inhibited integration into the labour market and social life in Canada. Participants sought employment in the Accommodation and Food Services (AFS) sector because they were unable to find employment in their fields of expertise. The sector was noted to have fewer barriers related to credentials and experience. Language was a significant barrier to over half of the women we spoke to; they identified language barriers as a reason to work in AFS.

I have to do a lot of studies here to be as the teacher or teacher assistant [her prior profession]. Yeah. But when I go to a restaurant I like this career of job, of work. And I’m mostly comfortable with this. [HA]

Participants worked part-time to learn English and obtain Canadian credentials in their field of expertise, experience, or interest. A 66-year-old participant noted how she felt like she was starting her life from “zero” working in a hotel while studying to get certified as an accountant in Canada [RD]. She had a master’s degree from her home country where she had practiced as a senior accountant. Participants with young children were further marginalized because of their caregiving work.

Women in Canada spend more time than men on unpaid work, with limited accessible and affordable childcare services contributing to women seeking work that is part-time and flexible [31, 48]. While childcare by relatives is reported as the second most used childcare arrangement in Canada [48], this is not an option for many recent immigrants; none of the women we interviewed had extended family in Canada. Most participants depended on the social support of their immediate nuclear family. Three single mothers did not have this support.

I was working before the pandemic five hours. I was on a part-time with a restaurant because I used to go to classes for two and a half hours in the morning. Then I would go home, prepare all the things for my kids to be at home and ready and then I would head down to the restaurant. [LG]

Community organizations moderated marginalization by offering spaces for socialization and community building, with some additionally offering free language and professional classes. However, not all the participants were aware of available support through such organizations.

Economic marginalization resulted from both limited incomes (with most participants earning minimum wages) and lack of intergenerational wealth. Participants spoke of losing everything they had to start a new in Canada and using life savings to supplement their minimum wage. Marriage did not provide much moderation as spouses faced similar challenges in the labour market. All participants earned an annual household income that was below Canada’s 2019 median income of C$ 62,000 after tax; they reported pre-pandemic annual incomes ranging from C$ 10,000 to C$ 56,000. This income is also below the 2018 living wage in British Columbia’s Metro Vancouver Regional District, where some participants resided, of C$ 76,112 annual income per household [49]. Social and economic marginalization, further exacerbated by the pandemic, significantly limited access to potential moderators of work precarity.

### Economic conditions and policies during COVID-19

Our analysis, presented in Table 1 below, suggests that the AFS sector coped with the market effects of COVID-19 by decreasing its full-time workforce and increasing its part-time workforce.

From March 2020, the government rolled out financial relief programs offering up to C$2,000 per month to eligible individuals who lost income because of the pandemic. To some participants, the benefit was equivalent to or higher than what they earned working part-time at minimum wage. This financial aid had immediate and long-term benefits. A single mother of two who was struggling to secure employment after being laid off, noted how these benefits enabled her to afford housing. Another woman, who previously found little time to study, used the break from work to complete her Canadian accounting credentials and was, at the time of the interview, applying for accounting jobs. Another single mother used part of the benefits to invite her mother to help her care for her child so that she could go seek more lucrative employment.

However, to some the government benefits were inaccessible or insufficient. Eligibility criteria tied to prior annual income, for instance, excluded new immigrants. The benefits also did not subsidize reduced incomes due to reduced work hours. Further, eligibility was tied losing employment through layoffs, hence workers could not resign from their work in the hope of accessing benefits.

The challenge for the CRB [government benefit program], when I resumed work back to 60 or 70 percent, so it already not eligible for the CRB because they have a criteria that you can only apply if you lost 50 percent of your salary. So, for that one, I was not able to apply for the CRB again when I resumed to work. [CH]

Knowledge, language, and technical barriers also limited access to government benefits. Four of the 11 women we interviewed did not know about the main government pandemic benefit program. Some of those who applied for the benefit depended on their children, husbands, friends, social workers, and an accountant for assistance.

When I had to open the link herself. I had to ask somebody to translate for me, to understand all the information. It needed really a lot of time to understand myself. Because when I was asking many people, there were many people saying, ‘oh you cannot apply’, ‘yes, you can apply’. So, I had to go through it and go through these challenges and ask somebody to translate for me. [LA]

Some of those who received government benefits found it insufficient in moderating their precarity from work. This inadequacy, however, was observably dependent on the unique living situation of the women including family size, childcare needs, and area of residence. A single mother of a 15-year-old, for instance, noted that C$2,000 from government benefit was “too much” to a point where she was able to make savings [RD]. However, this same amount was insufficient to women who had younger or more children. A mother of two with a husband who was precariously employed noted how with accommodation costs of over C$2,000 per month, the benefits were insufficient. Even with its limitations, government benefits moderated work precarity, but uncertainty around the timelines of these benefits made it an unreliable moderator. Participants expressed anxiety in not understanding program adjustments including when the benefits would end.

### Potential moderators and precarious work

Immigrant women described limited access to moderators related to social support services and networks. Those who immigrated into Canada during the pandemic did not benefit from prior social networks. During the pandemic, community resources became limited or inaccessible, and socialization opportunities were abruptly disrupted. Participants who took English classes noted that the shift to online learning limited opportunity to practice their English, delaying in their plans to advance in their careers. Physical distancing policies limited opportunities to draw on what social supports women had developed. A single mother, for instance, had previously depended on neighbours for childcare when she went to work but she was unable to depend on this support network during the pandemic because of fear of COVID-19 infection. She was to return to work because she could not afford formal childcare services, even with childcare centres opened following closure during the first few months of the pandemic.

I used to work and then after I said, no, I can’t sacrifice just my son because there is not anyone who will take care of him. I always give him to neighbours. They really help me it’s not safe for my child to be babysit with more people. Like today’s this one, today’s this one, so it’s really affected me mentally, emotionally. So I said, ‘OK, I will stop, and then I will wait for God’s grace, if COVID comes down, and then I will go back to work’….It [formal childcare] is expensive. Because I just – for me I say, ‘OK. Yes. You know? Let me just take this risk so my mum can come’. Because even if I’m working, I will be paying daycare. [with] Which paycheck? It’s not easy. [FM]

Limited access to financial resources was reported by all the women we interviewed. Workers in the AFS sector are paid the lowest average hourly wages compared to all other sectors in the Canadian economy. In 2021, this stood at C$17.35 an hour, C$12.68 less than the average hourly wage across other sectors [50]. This hourly wage is below the living wage in many cities across the country.^2^ In two cities where most of the immigrant women we interviewed reside, the 2021 living hourly wage was C$20.52 (Metro Vancouver) and $20.46 (Victoria) [51]. Wage distribution in the sector is also differential. In 2020, the average hourly wage in accommodation services subsector in was C$19.43 while that in food services subsector was C$16.33 [52]. Women in the sector earn less wages with a gender wage gap of nine percent nationally, based on median weekly wage [50]. Further, hiring discrimination in some food services establishments result in racialized and immigrant workers being in low wage positions such as dishwashing noted to be “the hardest job and the lowest paid job” [Key informant].

Women’s experiences of precarious work were structured by norms with the AFS sector. Less than five percent of workers in the AFS sector are members of a union and/or covered by a collective agreement, compared to 30.9 percent of workers across all sectors [53]. None of the sector workers we spoke to were unionized. Key informants highlighted the following challenges to collective bargaining rights: high turnover in the sector, push back by employers, and fear of retaliation. Employer-sponsored Temporary Foreign Workers were noted to be particularly vulnerable to limited workplace right because of their dependence on their employers for other aspects of the lives such as legal status, housing, and medical coverage [20]. The AFS sector employs the highest proportion of Temporary Foreign Workers (TFWs) among services-producing sectors [54]; between 2005 and 2012, the number of TFWs in the AFS sector increased by 926% [55].

Key informants noted that while unionized workers are more likely to have a formal contract of employment, such contracts were not “ironclad”: full-time workers can involuntarily be turned to part-time workers, a situation exacerbated by the pandemic. The food services subsector was characterized as “the wild wild west” as most establishments do not have human resources policies or protections against bullying or harassments [Key informant]; also see Hancey 2019 [56]. Where legal protections exist, key informants lamented about limited enforcement: The process of filing complaints was noted to be tedious and often met by victim-blaming practices. Employment insecurity in the sector further makes workplace protections and rights out of reach for workers who fear retaliation for taking action to redress workplace rights abuse.

### Work precarity: precarity of, from, and at work

*Precarity of work* took the form of job insecurity, unpredictable hours and pay, and career or employment insecurity. Some employers responded to reduced demand for service during the pandemic by dividing available work among all employees. While this meant more workers would remain employed, their employment was made precarious. Work hours and wages became unpredictable. One woman highlighted the challenge of planning for family expenses with the uncertainty of whether you would earn C$1,500 or C$3,000 in a particular month.

Sometimes I just, like, said to my workplace that I want some more hours and they were like, oh, we don’t have it to give it to you because we are, like, not busy. We don’t have enough customers… So even if you want to work, you can start earning from, like, 1,500 to 3,000 or, like, whatever, if you get more hours. Because, you know, the restaurant orders, like, 11 hours a day, but they will only give you five hours, four hours. [SS]

*Precarity from work* due to inadequate income was experienced by most participants. They constantly struggled to meet basic needs of their family earning minimum wage. Economic impacts of the COVID-19 pandemic, such as reduced work hours, further affected their ability to meet needs. Housing insecurity was highlighted as an important consequence of precarity from work. In major cities in British Columbia, the “average rent is not affordable for a single average earner without another source of income, even if they work full time”; the pandemic further deteriorating rental affordability [57] (p.10).

Before the COVID I used to work seven days a week and it was easy for me to pay my rent and pay my expenses. It was totally, like, fine with me financially… And when they told us to return back to work for these two days, it was, like, the whole day less than 150 dollars a day. Like, the two checks for the month, less than 500 dollars…I was always worried about is the rent. Like, even not the food. Not the transportation. Like, just the rent is the most worrisome for me. [NI]

While the provincial government placed a temporary ban on evictions and freeze on rent increase for existing tenants, participants indicated that some landlords threatened to increase rent or forced tenants to vacate in order to increase rent with the turnover to new tenants. Two women who were forced to move apartments during the pandemic evidenced how increase in rent and reduced incomes were intricately linked in compromising their ability to meet basic needs: One of them experienced a 45 percent increase in rent. At the time of the interview, one participant was looking for alternative accommodation as her rental cost became untenable. She was considering moving away from her community for lower rental costs, risking further social marginalization.

*Precarity at work* related to experiences of physical, psychological, and relational unsafety in the workplace. Increased workload due to additional cleaning protocols and engagement with clients was noted to put workers at a greater risk of contracting COVID-19. To protect herself, one participant used hand sanitizer excessively and developed an allergic reaction where her skin started peeling.

Even when the hall is very clean and tidy, your manager will say you have to go– after every 15 minutes you have to wipe all the tables; take out all the garbage because it has been 15 minutes and maybe it’s because people have sat there. So, you may contract COVID-19. So, you have to just keep on doing that. The whole day we are just doing that. [SSI]

Participants shared that taking time off work for a COVID-19 test or to stay home if sick translated to lost income as this time was not compensated. Testing clinics were far from participants’ workplaces translating to more unpaid time where tests were made mandated. This deterred some workers from informing their employers when feeling unwell.

Even though you have a regular fever, a regular cough, you can’t say then that I have cough. They will say, ‘OK, go [for a COVID-19 test]– just, it’s a long process. Like, you have to go very far…[SSI]

This, and another woman’s narration of two incidents where she was forced to take COVID-19 tests without pay, are indicative of struggles of AFS sector workers during the pandemic. Narrating one of several “uncomfortable situations” at work, a participant shared how a customer accused her of having COVID-19 because of coughing and demanded to see her manager [NI]. Such harassment by customers not only increased psychological and unsafety at work, but also had implications to workers’ income and job security at a time when many in the sector were losing their employment or work hours.

The woman quoted above also noted that the shift of healthcare from in-person to virtual services, combined with income loss associated with seeking care, further limited access. With fewer clinics open, she had to travel further to see a doctor and get prescription medication.

If you’re working anywhere and you are working full time, you will get only two days [paid sick leave], which are – you will get to the doctor and get medicine there… So, I have to get a holiday from my work which is, like, a loss for me. Like, if there are in-person doctor and I can visit anywhere on, like, a drop-in [SSI]

### Outcomes

*Mental health* of the recent immigrant women we interviewed notably degraded during the pandemic. While the negative mental health outcome was mostly associated with work precarity, other effects of the pandemic such as social insolation, increased unpaid care, inability to continue with routine activities, and disruption of self- and professional- development plans also contributed. Social distancing implemented in response to the pandemic coupled by fear of COVID-19 infection narrowed spaces for social interaction and support with neighbours, friends, and acquaintances. This not only limited potential moderators to work precarity outcomes but also contributed to negative mental health outcomes.

I feel that I have been depressed. It has been really hard for me to learn English and even if I try, the way that I can express myself is not the way that I would want to. And to be by myself at home has caused that state of depression itself. [LG]

While it might be challenging to detangle mental health outcomes emanating from social ramifications of the pandemic, some participants drew a direct link of their deteriorating mental health to work precarity. Particularly, they noted the coupled effect of low and unpredictable incomes, and increased costs of living during the pandemic on mental stress and anxiety about their inability to meet basic needs. Housing insecurity was a notable concern elaborated as inability to consistently pay rent or predict if one would be able to pay the following month’s rent. For instance, a woman whose rent increased during the pandemic developed anxiety and depression:

On the 16th of every month, I feel, like, very, very stressed. I have to collect C$ 1,400 for the rent. And once the beginning of the new month, I can breathe a little bit…It [COVID] affected my health and my mental wellbeing. I started to take antidepressants. [NI]

Government benefits mediated mental health outcomes associated with work precarity for those who received the benefits. Conversely, uncertainty about what would happen if and when the benefits ended contributed to anxiety among some participants.

In the face of work precarity and subsequent negative outcomes, participants exhibited a *strong sense of self-efficacy and internal locus of control*. While admitting how challenging it was to settle into Canada and be self-sufficient, they had plans of how they can overcome these challenges and succeed; from taking language classes and related tests, to pursuing their professional training and finally pursuing a career of their dreams. There was a general understanding on how to work within structural constraints towards individual success. Employment in the AFS sector was a bridge, a way for them to sustain themselves as they actualise their plans and dreams. Participants also articulated an understanding of structural barriers to their success that were out of their control. For example, mothers articulated challenges with the childcare model that made it difficult for them to secure full-time employment.

We are struggling [economically]. Like, we know that it [success] will happen. So, we are just trying to just overcome from this [struggle]. That’s why we are not just doing a job; we are also studying here, and we have goals even though they are long-term goals, but we know that if–when we achieve those goals, we will get easy into money and to get a job here. Because, like, I don’t want to end up my life, like, in McDonald’s, working, like, after 12 years. [SSI]

Self-efficacy was observable in how the women coped with work precarity and associated outcomes. Some coped by living frugally and ensuring their budget was “under control” [SO]; seeking community resources such as food banks; finding ways to supplement reduced incomes; exiting the AFS sector to seek more meaningful work; and seeking transnational support from networks in their home countries. One woman narrated starting a small catering business at home not only to supplement her income but also to moderate degrading mental health of her family. Those who tapped into their transnational networks (such as a single woman who invited her mother from North Africa to support her when she fell into depression) demonstrate resourcefulness at a time of isolation, when their limited circle of support in Canada was broken.

My children were staying at home all the time doing nothing and they’re being isolated. And that was one of the reasons that I decided to do my small business and catering from home. Like, to make good use of my time. And in order, like, not for me as well to get depressed. [LA]

Some women even refused to seek government and community aid. These women had previously never relied on such support and did not perceive challenges they faced to meeting basic needs as being out of their control. A single mother, who previously had a successful professional job in her home country, expressed this strong sense of control: She also saw this attribute as a virtue for her son to emulate.

I have the brave; I can do all the things well. So, especially I [don’t] want my son to know his mum just gets the government to fund, to live in Canada. So, I stopped it [taking government benefits]. I think–I just thought to myself I can study harder. I can work harder… I can live well just working hard. Not to stay at home, get the benefits. [RD]

## Discussion

This paper adds a lived experience perspective to the literature on pandemic precarities and the social determinants of health. The accounts included here demonstrate how the pre-existing social and economic marginalization was exacerbated by pandemic response policies, which in turn restricted the ability of recent immigrant women to moderate the outcomes of increasingly precarious work. Findings are reflective of those in other studies that also described how the pandemic did not create but rather exacerbated pre-existing precarious work conditions and work precarity [58, 59]. However, we further link this exacerbated precarity with the social determinants of health. Outcomes include direct health effects, such as decreased mental health, which have also been highlighted as a consequence of unemployment during the pandemic [60] and social isolation [61, 62]. Our findings further point to secondary health effects, such as limited access to testing and paid sick leave, which in turn increases risk of transmission and serious illness – potentially having negative effects on the public health response more broadly. Through applying the Work Precarity Framework we are able to map intersections between work precarity and other social determinants of health, such as access to income support, housing and education.

Notably, however, although the Work Precarity Framework associates work precarity to lower self-esteem and self-efficacy, we did not observe this as an outcome of work precarity among the recent immigrant women. Their understanding of structural barriers to their success was coupled by a strong sense of self-efficacy evidenced in how they coped and pursued long-term professional goals. In their publication ‘the hidden work of challenging precarity’, Mirchandani and Hande [63] indeed highlight that those who experience precarity are not passive victims but rather active agents with a sense of positive self-worth as they devise ways to cope. This suggests the need to build in strength-based approaches to the study of precarity.

The work precarity framework was useful in identifying the role of economic policies and moderators in structuring precarity. During the COVID-19 pandemic, while there was little to shield the recent immigrant women from work precarity, government pandemic benefit programs offered some relief. However, the government programs were inaccessible to some because of exclusionary eligibility criteria, and knowledge, language, and technical barriers. Additionally, the benefits’ inadequacy and uncertainty produced its own work precarity illustrating how social policies also play a prominent role in producing, reproducing, and distributing socio-economic marginalization and consequently work precarity. Further, inadequate investments in childcare created opportunity costs, structured by social gender norms assigning women as primary caregivers and associated with time spent on unpaid work vis-à-vis paid work [31]. Immigrant women who have little or no social support and family in Canada face additional barriers to accessing informal childcare. Among the women we interviewed, therefore, mothers and particularly single mothers faced additional socio-economic marginalization.

Our study offers learnings to inform a more equitable pandemic response and recovery, that mitigates as opposed to exacerbates the social determinants of health. Government benefit programs have the potential of moderating work precarity among marginalized groups in times of crisis if barriers to access are addressed. Specifically, eligibility criteria should be made inclusionary, and knowledge, language, and technical barriers could be addressed in collaboration with existing community networks. Supporting community organizations through consistent funding could also bridge access to resources for recent immigrants. Bambra et al. [60] highlight the importance of welfare policies in protecting vulnerable populations against structural pressures to increase inequalities in prior financial crisis. Nonetheless, while these charitable avenues moderate experiences and outcomes of work precarity, recent immigrant women would benefit more from structural changes which would increase their opportunities in the Canadian labour market, reduce precarious work, and reduce their cost of living.

Longstanding policy issues contributing to unaffordable childcare and inaccessibility of the labour market to immigrants should particularly be prioritized. Specifically, the B.C. provincial government can adopt policies recognizing international professional qualifications and experience: This would reduce economic marginalization which drives immigrant workers into precarious employment. While the government expanded ten-dollar-a-day childcare [64], these options remain largely inaccessible: Further expansion should prioritize lone and low-income parents. The government should also work towards bridging the gap between minimum wage and living wage, and closing the gender wage gap. Additionally, existing precarious work characterized by variable work hours and incomes in the accommodation and food services sector should be addressed. Pandemic risk should not squarely fall on workers through labour flexibilization. The government should put in place policies that protect workers against economic downturn and industry response resulting from pandemics and other emergencies, including benefit relief programs and enforceable industry-level recall rights tied to prior work conditions. Recognizing the role of policies in producing, reproducing, and distributing socio-economic marginalization, and by extension work precarity, efforts should be made to protect those already marginalized.

While this study does not attempt to test or validate the Work Precarity Framework, it does highlight its utility. The study also reveals the importance of researchers familiarizing themselves with specific processes and structures contributing to precarity among their research participants. Listed social and economic marginalization, economic conditions and policies, and potential moderators, for instance, are not exhaustive; hence, they should be considered entry points to further inquiry. Utilization of the framework as an analytical tool in research with immigrant workers would, for instance, also consider marginalization that transcend national boundaries including international gendered division of labour which shape national policies such as temporary foreign worker programs. In the Canadian context, for instance, such programs target migrant workers based on gender and nationality for specific roles in the labour market. A closer look into such processes and marginalization they produce would support an intersectional analysis of lived experiences among this group of workers. Our research also found that a strong sense of commitment to success and subsequent future planning, and an understanding of structural barriers limiting their capacity acted as important potential moderators to negative psychological outcomes for immigrant workers.

## Conclusion

This paper illuminates how pre-existing and ongoing marginalization are reproduced during a health crisis particularly for those at the intersection of gender, race, migration, and labour inequities. It complements and adds to the growing body of work on precarity as a social determinant of health and on pandemic precarities, particularly by applying, and suggesting further developments to, the Work Precarity Framework. We also offer policy directions primarily aimed at addressing socio-economic marginalization contributing to work precarity, for a more equitable pandemic response and recovery.

As a relatively small qualitative study, this research does not aim to be representative of or generalizable to all immigrant women working in the AFS sector. Instead, it aims to share the lived experiences and perspectives of a group of women highly effected by both the pandemic and precarity, recognizing that experience is knowledge well positioned to inform equity-based recovery. Additional research employing mixed or quantitative methods and with other highly effected populations, perhaps also using the Work Precarity Framework, would add to the discussion this paper initiates.

## Data Availability

The dataset used and/or analysed during the current study available from the corresponding author on reasonable request.

## Footnotes

1 One scheduled interview was cancelled because the participant ended up being unavailable because of double-booking her time. She expressed her desire to participate in the research, but her schedule was very busy. We were unable to reschedule. During the weekdays, she would go for English classes in the morning before going back home to prepare her children for school; she would then go back home to prepare herself for her part-time work which lasted until end of school; she would then take her children to after-school activities; then come home to make then dinner then prepare for the next day. Over Saturdays, she cared for her children and would take them for sport activities. On Sundays, she was also not available as she was in church for most of the day. This schedule was similar to other women we interviewed.

2 The living wage is calculated based on a four-member household with two children, financing “a bare bones budget…to pay for necessities, support the healthy development of their children, escape severe financial stress and participate in social, civic and cultural lives of their communities” (Ivanova et al., 2018, 1,3).
